# Azithromycin Differentially Alters TCR-Activated Helper T Cell Subset Phenotype and Effector Function

**DOI:** 10.3389/fimmu.2020.556579

**Published:** 2020-09-30

**Authors:** Abdul Wahid Ansari, Fatemeh Saheb Sharif-Askari, Manju Nidagodu Jayakumar, Abdul Khader Mohammed, Narjes Saheb Sharif-Askari, Thenmozhi Venkatachalam, Bassam Mahboub, Reinhold E. Schmidt, Rifat Akram Hamoudi, Rabih Halwani, Qutayba Hamid

**Affiliations:** ^1^Sharjah Institute for Medical Research, University of Sharjah, Sharjah, United Arab Emirates; ^2^Department of Pulmonary Medicine, Rashid Hospital, Dubai Health Authority, Dubai, United Arab Emirates; ^3^Department of Clinical Immunology and Rheumatology, Hannover Medical School, Hanover, Germany; ^4^Department of Clinical Sciences, College of Medicine, University of Sharjah, Sharjah, United Arab Emirates; ^5^Division of Surgery and Interventional Science, University College London, London, United Kingdom; ^6^Prince Abdullah Ben Khaled Celiac Disease Chair, Department of Pediatrics, Faculty of Medicine, King Saud University, Riyadh, Saudi Arabia; ^7^Meakins-Christie Laboratories, Faculty of Medicine, McGill University, Montreal, QC, Canada

**Keywords:** azithromycin, CD4+ helper T cells, anti-inflammatory, CCR4, CXCR3, IFN-γ, IL-4, apoptosis

## Abstract

In addition to their antibiotic activities, azithromycin (AZM) exhibits anti-inflammatory effects in various respiratory diseases. One of the potent anti-inflammatory mechanisms is through inhibition of CD4+ helper T (Th) cell effector function. However, their impact on specific Th subset is obscure. Herein, we demonstrate the cellular basis of phenotypic and functional alterations associated with Th subsets following AZM treatment *in vitro*. Using well-characterized Th subset specific chemokine receptors, we report significant suppression of T cell receptor (TCR)-stimulated hyperactivated CCR4+CXCR3+ (Th0) expansion compared to CCR4-CXCR3+ (Th1-like) and CCR4+CXCR3- (Th2-like) cells. Interestingly, this effect was associated with diminished cell proliferation. Furthermore, AZM significantly inhibited the inflammatory cytokines IFN-γ and IL-4 production, CCR4 and CXCR3 receptor expression, and viability of Th0, Th1-like, and Th2-like subsets. Our findings suggest that AZM differentially affects TCR-activated Th subsets phenotype and function, and CCR4 and CXCR3 downregulation and suppressed Th0 subset expansion could potentially influence their trafficking and differentiation into cytokine-producing effector cells.

## Introduction

Azithromycin (AZM) is a potent macrolide antibiotic used to treat various respiratory diseases. Prophylactic treatment with low-dose azithromycin has shown clinical benefits by reducing exacerbations in COPD (1–3), asthma (4, 5), cystic fibrosis (6), and non-cystic fibrosis bronchiectasis (7, 8) and mycobacterial infections (9). In addition to their antimicrobial properties, AZM is known to have numerous anti-inflammatory and immunomodulatory effects. Several studies have supported the anti-inflammatory effects of AZM on structural and immune cells (10). Immunomodulatory effects of AZM have also been reported in a recent study on an animal model where treatment resulted in clearance of *Bordetella pertussis* infection (11). The majority of earlier studies have focused on innate immune cells, mainly monocytes, in context to pro-inflammatory cytokines including IL-1β, TNF-α, and IL8 (12–15). Given the contribution of CD4+ helper T cells in various respiratory diseases, efforts have been made to understand the immunomodulatory effects of AZM on these cells. Recent studies have shown suppression of CD4+ T cell effector function following AZM treatment potentially by inhibiting proliferation and inducing cell death (10, 16). Mechanistically these effects are believed to be due to inhibition of mammalian target of Rapamycin (mTOR) activity (16), involvement of FasL-Fas pathway, and down-regulation of anti-apoptotic protein Bcl-xL (17).

Naive CD4+ T cell recognition of antigens *in vivo* and polyclonal TCR stimulation *in vitro* result in cell expansion, differentiation, and effector cytokine production. This changes the dynamicity of chemokine receptor expression pattern on T helper (Th) cells (18, 19). Th1 and Th2 are the most characterized and studied Th subsets (20–22) and are identified by their ability to produce signature cytokines such as IFN-γ (Th1) and IL-4, IL-5, and IL-13 (Th2), respectively. The balance between these cells plays a critical role in disease pathogenesis as well as their outcome. Contribution of Th1 and Th2 cytokines have been implicated in the lung infection and chronic inflammation including allergic asthma. In addition to cytokine production, Th1 and Th2 cells upon TCR stimulation acquire different migratory capacities by expressing differential chemokine receptors (19, 23) on both polarized and un-polarized cells. For example, Th1 cells predominantly express CXC chemokine receptor 3 (CXCR3) and CCR5 (24–26) while Th2 are known to express CCR3 (27), CCR4 (24, 25, 28), and CCR8 (29). After acquiring chemokine receptors, these effector cells then migrate to the site of inflammation in the peripheral tissues.

The previous studies have shown the immunomodulatory effects of AZM on bulk CD4+ T cells (10, 16). Given the heterogeneity of helper T cells, it is worth understanding the impact of AZM on Th subsets to delineate their effector function at cellular level. In this study, using well-characterized Th1 and Th2 subset specific chemokine receptors CCR4 and CXCR3, respectively, we identified CCR4+CXCR3+ (Th0), CCR4-CXCR3+ (Th1-like), CCR4+CXCR3- (Th2-like), and CCR4-CXCR3- (DN) subsets in peripheral blood CD4+ T cells. The subset nomenclature is purely based on previous studies (18, 19). In this study we report the cellular basis of phenotypic and functional changes associated with AZM treatment of various Th subsets.

## Materials and Methods

### Human CD4+ T Cell Enrichment and Stimulation

Peripheral blood mononuclear cells (PBMCs) were isolated from freshly drawn blood of healthy volunteers using histopaque (Sigma-Aldrich) gradient centrifugation. CD4+ T cell enrichment was performed using EasySep human CD4+ T cell isolation kit (Stem Cell Technologies, United States). Cells were routinely checked for purity of above 96% as detected by flow cytometry using anti-CD3-AlexaFluor 700 (clone OKT3) and anti-CD4-eFluor 780 (clone OKT4, eBiosciences, United States) monoclonal antibodies (mAbs). Briefly, 5 × 10^5^ cells were activated with plate-bound anti-CD3 (clone UCHT1, 4 μg/ml) and soluble anti-CD28 (clone CD28.2, 2 μg/ml, eBiosciences, United States) antibodies in a 24-well culture plate for 3 days. All cell culture experiments were performed in complete RPMI 1640 (Sigma-Aldrich) supplemented with 10% fetal bovine serum (FBS, Sigma-Aldrich).

### FACS Sorting of Helper T Cell Subsets

In some experiments fluorescence-activated cell sorting (FACS) was performed to isolate CCR4+CXCR3- (Th2-like), CCR4+CXCR3+ (Th0), and CCR4- CXCR3+ (Th1-like) cells. Briefly, PBMCs were stained with anti-CD3-AlexaFluor 700 (clone OKT3), anti-CD4-eFluor 780 (clone OKT4), anti-CCR4-AlexaFluor 647 (clone L291 H4), and anti-CXCR3-PECy7 (clone CEW330) as described above. Sorting was performed at BD FACS Aria III (BD Biosciences, United States) using BD FACS Diva software. A purity of >94% percent was achieved during routine testing.

### Cell Proliferation Assay

Enriched CD4+ T cells were labeled with 2.5 μM carboxyfluorescein succinimidyl ester (CFSE, Invitrogen, United States) for 8 min at room temperature in dark with intermittent mixing. Cells were washed twice with 10 ml of ice-cold complete RPMI 1640 medium and stimulated with immobilized anti-CD3 and soluble anti-CD28 antibodies in presence or absence of azithromycin (Sigma-Aldrich). Cells were harvested on day 3, washed with phosphate buffered saline (PBS, Sigma-Aldrich), and stained with anti-CCR4-AlexaFluor 647 (clone L291 H4, Biolegend, United States) and anti-CXCR3-PECy7 (clone CEW330, eBiosciences, United States) mAbs in FACS staining buffer for 25 min at 4°C. Washed cells were acquired with flow cytometer (BD FACS Aria III, BD Biosciences, United States) using BD FACS Diva software. Single stain cells were used for flow compensation.

### Apoptosis Assay

CD4+ T cells were stimulated with anti-CD3 and CD28 in presence or absence of indicated dose of AZM. Cells were harvested on day 3 and stained with anti-CCR4-AlexaFluor 647 and anti-CXCR3-PECy7 mAbs. Staining was performed with PE-Annexin-V and 7-AAD Detection kit (Biolegend, United States) according to the manufacturer’s instructions. Apoptosis was detected with flow cytometer. In some experiments FACS-sorted Th0, Th1-like, and Th2-like cells were also tested for apoptosis using the procedure described above.

### Intracellular Cytokine Detection

Anti-CD3 and anti-CD28 stimulated cells were cultured in presence and absence of AZM for 3 days. Brefeldin A, 10 μg/ml (eBiosciences, United States) was added in the last 5 h of the culture. Cells were harvested and surface stained with anti-CCR4-AlexaFluor 647 and anti-CXCR3-PECy7 monoclonal antibodies followed by intracellular cytokine staining using Cytofix/CytoPerm kit (BD Biosciences, United States) as per manufacturer’s instructions. Anti-IFN-γ-APC EF780 (clone 4S.83) and anti-IL-4-PE (clone 8D4-8, eBiosciences, United States) mAbs were used to detect intracellular cytokine production. Stained cells were acquired by flow cytometer and analyzed using appropriated gating.

### Sorted Th Subset IFN-γ and IL-4 Cytokine Detection by ELISA

Around 3–5 × 10^4^ FACS sorted Th subsets were stimulated as described above with plate-bound anti-CD3 and soluble anti-CD28 in a 48-well culture plate in presence or absence of indicated concentration of AZM. Culture supernatants were harvested on day 3 and stored at −20°C until use. ELISAs were performed using human DuoSet IFN-γ and IL-4 ELISA kits (R&D Biosystems) according to manufacturer’s instructions.

### Transwell Chemotaxis Assay

Chemotaxis was performed using a 24-well, Transwell plate (8.0-μm pore size; Corning, Corning, NY, United States) as described previously (30). Briefly, TCR-stimulated CD4+ T cells culture in presence or absence of AZM were harvested on day 3. Cells were washed with serum-free RPMI 1640 medium and adjusted to a density of 5 × 10^5^ cells/ml in serum free RPMI 1640 medium. Medium alone or supplemented with indicated concentration of CCL22 or CXCL11 chemokines was placed in the lower chamber of the Transwell, and 100 ul cells suspension was loaded onto upper chamber. Culture plate was incubated for 120 min in a 5% CO_2_-humidified incubator at 37°C. Numbers of cells migrating to the lower chamber were counted under a microscope using a hemocytometer.

### Ethics Statement

This study was approved by Ethics Committee of Dubai Health Authority (DHA) and Dubai Scientific Research Ethics Committee (DSREC-03/2019_10) United Arab Emirates. Written informed consents were obtained from each participants and study was conducted according to Helsinki Declaration.

### Statistical Analysis

For statistical evaluation of data, we have used GraphPad PRISM (v.5.0) software. One-way analysis of variance (ANOVA) followed by Tukey’s multiple comparison test to compare more than two groups. For chemotaxis assay unpaired *t*-test was used for analyzing two groups, and *p*-value of <0.05 were considered significant.

## Results

### AZM Suppresses Expansion of TCR-Induced Th Subsets

**FIGURE 1:**
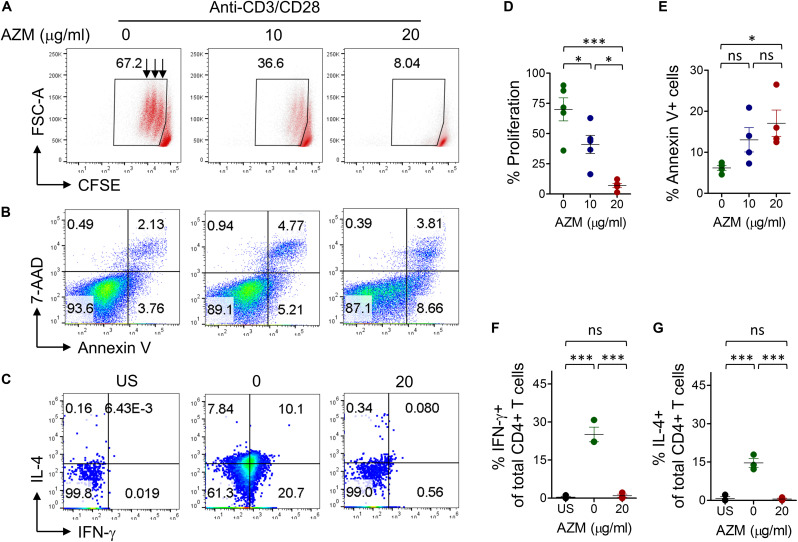
AZM suppresses bulk CD4+ T cells function. **(A)** *Cell proliferation.* CFSE-labeled purified CD4+ T cells were stimulated with anti-CD3 anti-CD28 in presence of indicated concentration of AZM as described in materials and methods. Live cells were gated to detect level of proliferation. Representative FACS plots show the percent CFSE+ cells and each streak in gated population represents a cell division. **(D)** Aligned dot plots show mean percentage ± SEM cell proliferation. Data shown are from five healthy individuals. **(B)** *Cell viability.* Anti-CD3/CD28 stimulated cells were treated with indicated concentration of AZM. On day 3 cell death assay was performed using Annexin V and 7-AAD labeling. Representative FACS plots show percent apoptosis. **(E)** Aligned dot plots show mean ± SEM of total Annexin V+ (apoptosis) cells. Data shown are from four healthy individuals. **(C)** *Cytokine production.* Anti-CD3/CD28 stimulated and unstimulated (US) cells were double-stained with anti-IFN-γ and anti-IL-4 mAbs as described in the section “Materials and Methods.” FACS plots show the percent cytokine production by AZM treated and untreated cells. Cells without anti-CD3 and CD28 stimulation were taken as control (US). Aligned dot plots show mean ± SEM of total IFN-γ+ **(F)** and IL-4+ cells **(G)**. Data shown are from three healthy individuals. *P < 0.05, **P < 0.01, ***P < 0.001, ns stands for non-significant.

TCR stimulation is known to trigger CD4+ T cells cell activation, expansion, and cytokine production. At first instance, we attempted to understand the effect of AZM on bulk CD4+ T cells. Enriched CD4+ T cells with purity of above 95% (Supplementary Figure S1A) were stimulated with anti-CD3 and CD28 in presence or absence of various doses of AZM for 3 days. CFSE-labeled cells were cultured in the above condition and proliferation was detected by flow cytometer. We observed significant inhibition in cell proliferation (Figures 1A,D) with greater effect of AZM at 20 μg/ml (*P* < 0.001). Interestingly, in absence of AZM, the proliferative cells undergo three rounds of cell division on day 3, and that progressively reduced to either two or one division with increasing doses of AZM. To understand potential mechanism of proliferation inhibition, we performed cell death assay on TCR stimulated cells. AZM treated cells undergo significant apoptosis only at higher dose (*P* < 0.05) as measured by Annexin V labeling (Figures 1B,E). To determine the CD4+ T cell effector function, intracellular cytokine staining was performed on day 3 of culture in presence and absence of 20 μg/ml AZM. A massive reduction in IFN-γ (*P* < 0.001) and IL-4 (*P* < 0.001) production was observed following AZM treatment (Figures 1C,F,G). Above observations in cell proliferation, cytokine production and apoptosis are in line with previous studies (10, 16).

**FIGURE 2:**
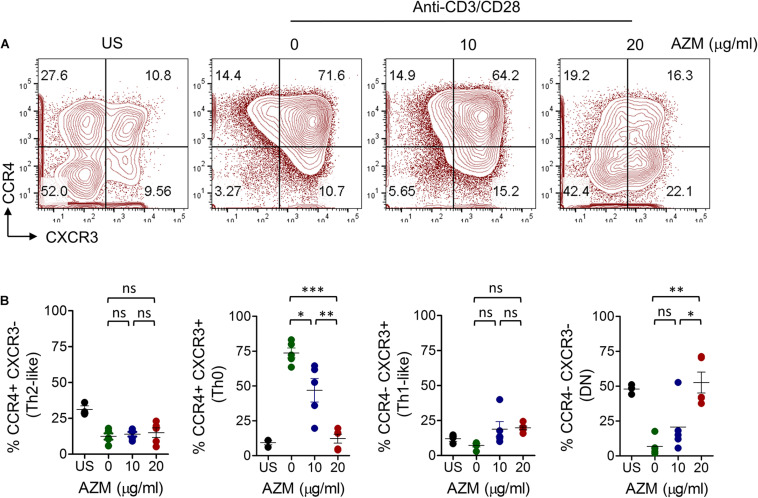
AZM inhibition of helper T cell subset expansion. CD4+ T cells were stimulated with plate-bound anti-CD3 and soluble anti-CD28 in presence of indicated concentration of AZM for 3 days. **(A)** Representative contour FACS plots showing percent frequencies of various Th subsets. **(B)** Aligned dot plots show means ± SEM of Th subsets, CCR4+CXCR3- (Th2-like), CCR4+CXCR3+ (Th0), CCR4-CXCR3+ (Th1-like), and CCR4-CXCR3- (DN) cells. Data presented are from five independent experiments performed on healthy individuals (*n* = 5). *P < 0.05, **P < 0.01, ***P < 0.001, ns stands for non-significant.

We then tested if AZM effects we observed on bulk CD4+ T cells differing at Th subset level. Enriched CD4+ T cells were stimulated under similar condition as mentioned above for 3 days. Cells were stained with chemokine receptor-specific anti-CCR4 and anti-CXCR3 mAbs and their frequencies were determined by flow cytometer. Four distinct cell populations CCR4-CXCR3+ (Th1-like), CCR4+CXCR3- (Th2-like), CCR4+CXCR3+ (Th0), and CCR4-CXCR3- (DN) were identified within resting CD4+ T cells (Supplementary Figure S1B). However, TCR stimulation resulted in expansion of Th0 subset. Of note, this expansion was drastically reduced by AZM in a dose dependent manner (Figures 2A,B). Nearly four-fold reduction in Th0 subset (*P* < 0.001) was found at a dose of 20 μg/ml compared to untreated cells. In contrast, DN subset showed a significant increase in cell proportion (*P* < 0.01).

**FIGURE 3:**
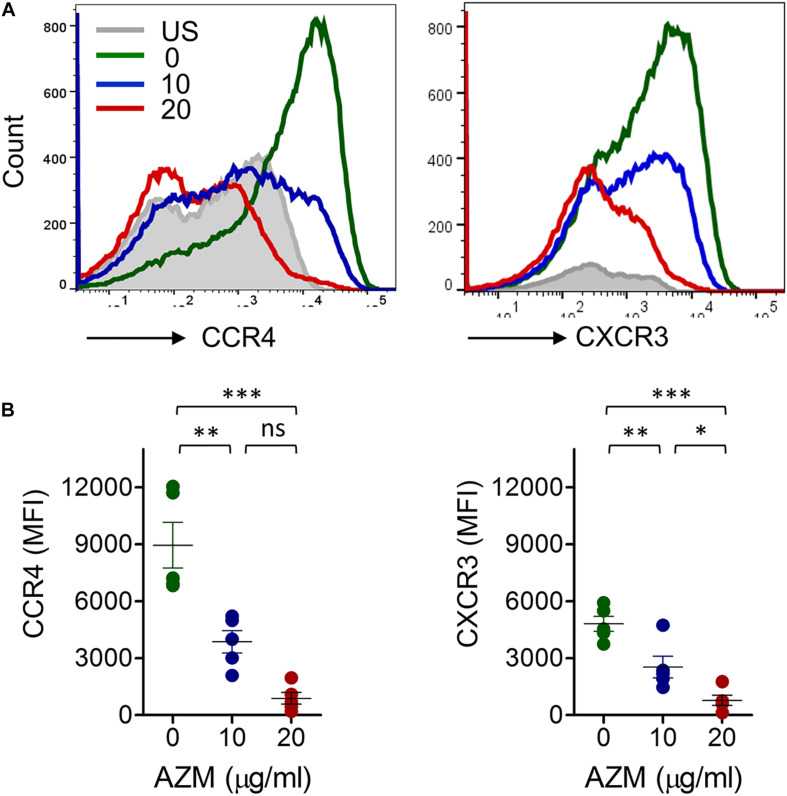
AZM downregulates CCR4 and CXCR3 expression on bulk CD4+ T cells. **(A)** Purified CD4+ T cells were stimulated with anti-CD3 and CD28 in presence of indicated concentration of AZM for 3 days. Cells were stained with anti-CCR4 and anti-CXCR3 monoclonal antibodies and looked for expression using flow cytometry. Representative FACS overlay histogram showing mean fluorescence intensities (MFI) of AZM treated and untreated cells. TCR unstimulated cells (US) serve as control. **(B)** Aligned dot plots show mean ± SEM of total CCR4+ and CXCR3+ cells on bulk CD4+ T cells. Data presented are from five independent experiments performed on healthy individuals (*n* = 5). *P < 0.05, **P < 0.01, ***P < 0.001, ns stands for non-significant.

We also tested the influence of AZM on CCR4 and CXCR3 receptor expression on a per cell basis. AZM treatment resulted in significant decline (*P* < 0.001) in the mean fluorescence intensity of both receptors in a dose dependent manner (Figures 3A,B). The downregulation of CCR4 is in agreement with previous reports where roxithromycin down modulated CCR4 expression on Th2 cells (31). These data suggest that AZM not only suppresses the cell expansion but chemokine receptor density as well. To understand the functional relevance of CCR4 and CXCR3 downregulation, chemotaxis assays were performed using Transwell system. AZM treated CD4+ T cells showed a general trend of reduced migration to CCR4 and CXCR3 ligands CCL22 and CXCL11, respectively (Supplementary Figures S2A,B).

### AZM Suppresses Proliferation of Th Subsets

**FIGURE 4:**
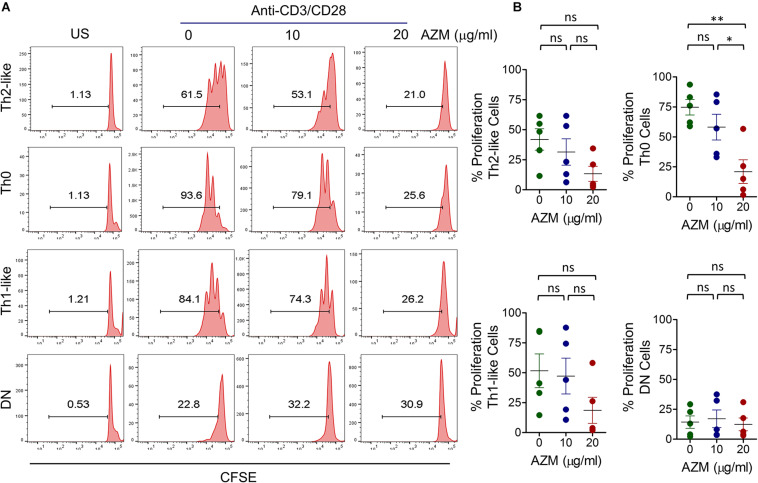
AZM suppresses helper T cell subset proliferation. CFSE-labeled CD4+ T cells were stimulated with anti-CD3 and anti-CD28 in presence of indicated concentration of AZM for 3 days. Cells without anti-CD3 and CD28 activation (US) were taken as control. Individual Th subsets (Supplementary Figure S1B) were gated to determine cell proliferation by flow cytometer. **(A)** Numbers in representative FACS histograms show percent proliferation based on CFSE dilution. **(B)** Aligned dot plots show mean ± SEM of CFSE dilution by different Th subsets. Data presented are from five independent experiments performed on healthy individuals (*n* = 5). *P < 0.05, **P < 0.01, ns stands for non-significant.

Next, we sought to determine the effects of AZM on Th subset proliferation. CFSE-labeled CD4+ T cells were stimulated with anti-CD3 and anti-CD28 in the presence or absence of various doses of AZM as described in the section “Materials and Methods.” We observed differential proliferative capacities of Th subsets with TCR stimulation as detected by CFSE dilution (Figure 4A). Of note, Th0 subset exhibited highest proliferation compared to others. Interestingly, when we examined the effect of AZM, only Th0 subset at a dose of 20 μg/ml showed a significant proliferative reduction (*P* < 0.01) compared to other subsets (Figure 4B). This suggests that TCR-stimulated hyperactivated Th0 subsets are more inclined to AZM mediated proliferative inhibition compared to the rest of the subsets.

### AZM Inhibits IFN-γ and IL-4 Cytokine Production by Th Subsets

**FIGURE 5:**
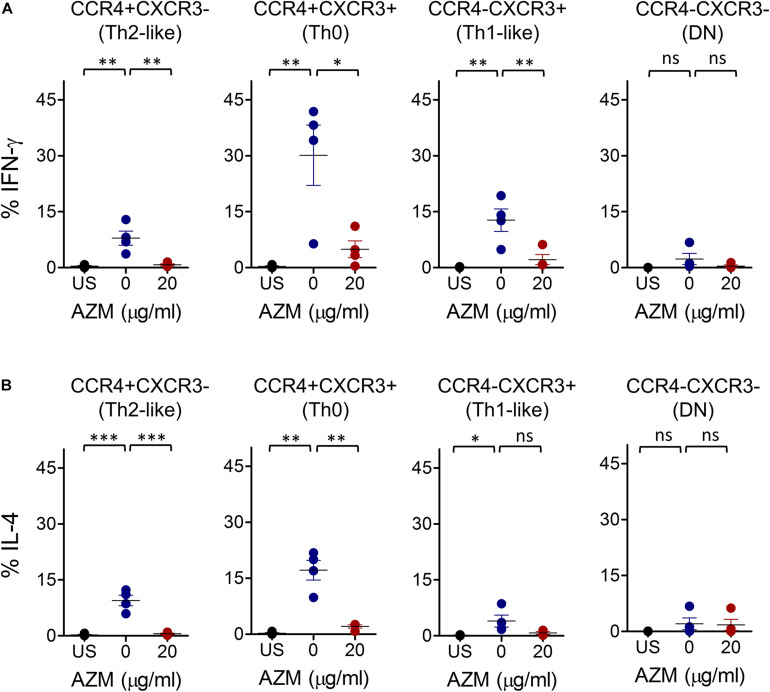
AZM inhibits IFN-γ and IL-4 production of helper T cell subsets. Anti-CD3 and CD28 stimulated cells were cultured at indicated concentration of AZM for 3-days. BFA was added in last 4 hr of the culture. Cells without anti-CD3/CD28 stimulation were taken as control (US). Intracellular cytokine staining was performed with anti-IFN-γ and anti- IL-4 mAbs and detected by flow cytometer. Th subset cytokine production was determined by gating on specific Th subsets. Aligned dot plots show mean ± SEM of total intracellular IFN-γ+ **(A)** and IL-4+ **(B)** production by each subset. Data presented are from four independent experiments performed on healthy individuals (*n* = 4). *P < 0.05, **P < 0.01, ***P < 0.001, ns stands for non-significant.

To determine the effects of AZM on Th subset effector function, we tested Th1- and Th2-associated inflammatory cytokine IFN-γ and IL-4 production. Given the significant effects of AZM on Th subset proliferation and apoptosis at 20 μg/ml, we performed intracellular cytokine staining (ICS) assays at same dose. TCR stimulated and unstimulated cells were stained with anti-IFN-γ and anti-IL-4 monoclonal antibodies and detected subsequently with flow cytometer. Various Th subset gated populations were examined for IFN-γ and IL-4 production as done for bulk CD4+ T cells (Figure 1C). TCR stimulation resulted in differential IFN-γ and IL-4 production by various Th subsets compared to unstimulated cells (Figures 5A,B). However, when we tested the influence of AZM, both IFN-γ and IL-4 expression were significantly reduced in all except DN subsets. Notably, the magnitude of IL-4 inhibition was much higher (*P* < 0.001) than IFN-γ (*P* < 0.01) suggesting a greater influence of AZM on Th2 cytokines. In addition, as control PMA/ionomycin stimulated cells also showed cytokine expression by various Th subsets (Supplementary Figure S5). Furthermore, to understand the effects of AZM on specific Th subsets, FACS-sorted (Supplementary Figure S3) and TCR-stimulated Th0 (CCR4+CXCR3+), Th1-like (CCR4- CXCR3 +), and Th2-like CCR4+CXCR3-) cells were cultured in presence or absence of AZM. ELISA results of culture supernatant obtained from two donors show severe inhibition of both IFN-γ and IL-4 cytokine production following AZM treatment (Supplementary Figures S4A,B). These data suggest that AZM not only inhibits cytokine production of bulk CD4+ T cells but also their purified subsets.

### AZM Promotes Apoptosis of Th Subsets

**FIGURE 6:**
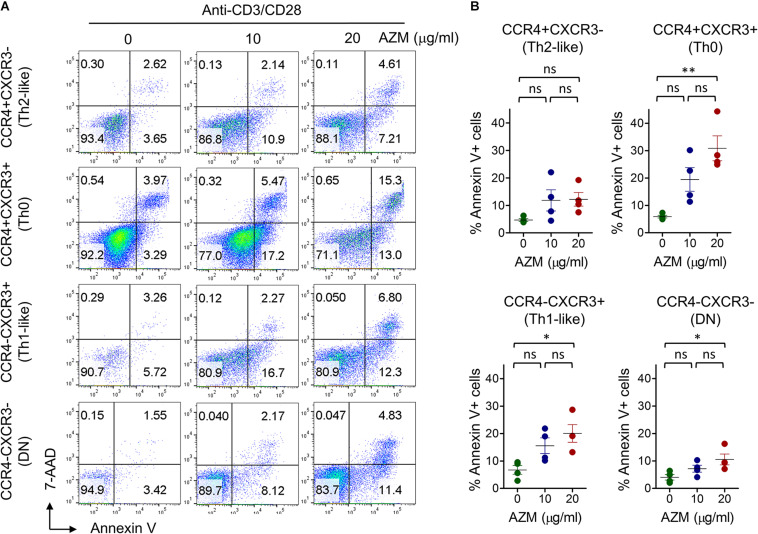
AZM induces helper T cell subset apoptosis. Anti-CD3 and CD28 stimulated cells were treated with indicated concentration of AZM. On day 3 cell death assay was performed using Annexin V and 7-ADD labeling. Unstimulated (US) cells were taken as control. **(A)** Representative FACS plots show percent apoptosis by each Th subset. **(B)** Aligned dot plots show mean ± SEM of total Annexin V+ (apoptosis) cells. Data presented are from four independent experiments performed on healthy individuals (*n* = 4). *P < 0.05, **P < 0.01 and, ns stands for non-significant.

**FIGURE 7:**
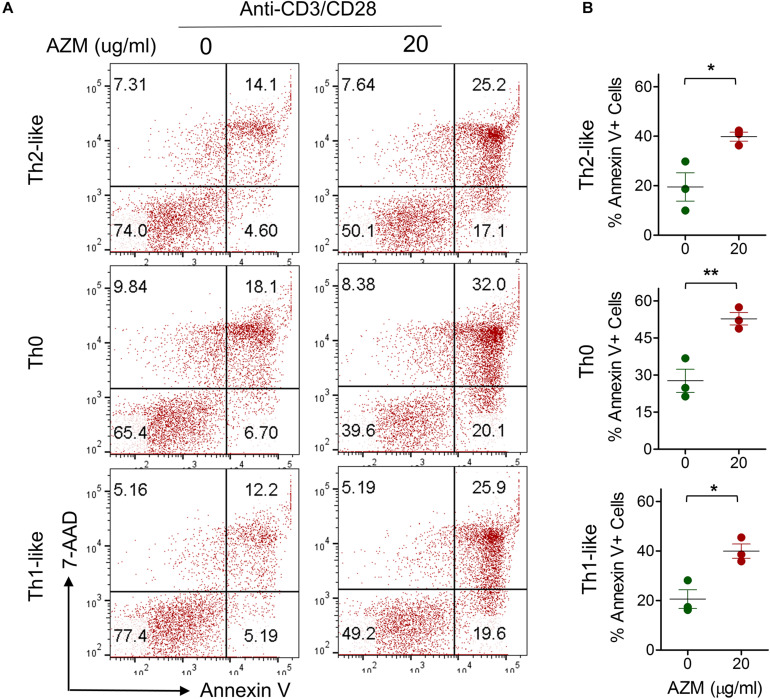
AZM induces apoptosis of FACS sorted Th subsets. Anti-CD3 and CD28 stimulated cells were treated with indicated concentration of AZM. **(A)** Representative FACS plots show percent apoptosis by Th2-like, Th0, and Th1-like subset. **(B)** Aligned dot plots show mean ± SEM of total Annexin V+ (apoptosis) cells. Data presented are from three independent experiments performed on healthy individuals (*n* = 3). *P < 0.05, **P < 0.01 and, ns stands for non-significant.

One of the mechanisms of AZM mediated inhibition of bulk CD4+ T cell effector function is through apoptotic induction (17). Similar effects were observed in our experiments on bulk CD4+ T cells (Figures 1B,E). To evaluate the apoptosis-inducing effect of AZM on various Th subsets, anti-CD3 and CD28 stimulated CD4+ T cells were cultured in presence or absence of AZM for 3 days. Cells were harvested and labeled with viability dye Annexin V and 7-AAD to estimate the level of cell death. Th subsets gated cells were looked at for total Annexin V positivity (Figures 6A,B). Flow cytometry data revealed significant apoptosis at 20 μg/ml in Th0 (*P* < 0.01), Th1-like (*P* < 0.05), DN (*P* < 0.05), but not in Th2-like (*P* > 0.05) subset. To further understand the apoptosis inducing capacity of AZM on specific Th subset, TCR-stimulated FACS-sorted Th0, Th1-like, and Th2-like cells were cultured in the presence or absence of AZM. We observed AZM mediated apoptosis in all the Th subsets, Th1-like (*P* < 0.05), Th2-like (*P* < 0.05), and Th0 (*P* < 0.01) (Figures 7A,B).

## Discussion

Given the heterogeneity and chemokine receptor dynamicity within Th cells, we sought to delineate the impact of AZM at the subset level. Herein, using well-characterized Th1 and Th2 subset specific chemokine receptor CXCR3 and CCR4, we show suppressed cell expansion, diminished proliferation associated with enhanced apoptosis, downregulation of CCR4 and CXCR3 expression, and inhibition of inflammatory cytokines IFN-γ and IL-4 by Th cells. These findings suggest the differential response of Th subsets toward AZM. We believe that AZM mediated Th subset alterations could potentially modulate Th0 cell differentiation into Th1 and Th2 cytokine-producing effector cells during inflammatory reactions.

At first instance, we investigated the effect of AZM on bulk CD4+ T cell effector function. We observed proliferative inhibition, diminished IFN-γ and IL-4 cytokine production, and apoptotic induction upon treatment with AZM. These findings are in line with previous studies on CD4+ T cells (10, 16). We also observed that in absence of AZM, the proliferative cells undergo three rounds of cell division on day 3 and that progressively reduced to either two or one division with increasing doses of AZM. These data clearly indicate that AZM inhibits cell proliferation by modulating cell cycle. In contrast to other studies where higher doses of AZM were used to induce apoptosis (10, 16), our results show significant CD4+ T cells apoptosis even at relatively lower dose (20 μg/ml). Polyclonal TCR stimulation resulted in selective Th0 subset expansion associated with declined proportions of Th1-like, Th2-like, and DN subsets as well as elevated levels of CCR4 and CXCR3 expression on CD4+ T cells. These observations support the notion that TCR stimulation up-regulate CCR4 and CXCR3 expression on activated CD4+ T lymphocytes (18, 19, 24). However, the elevated levels of CCR4 and CXCR3 expression were significantly downregulated with subsequent treatment of AZM. To understand the functional relevance of AZM mediated CCR4 and CXCR3 inhibition, our chemotaxis assays demonstrated reduced migration of CD4+ T cells in response to their cognate ligands CCL22 and CXCL11. That means AZM could confer anti-inflammatory activity potentially via inhibition of CCR4 and CXCR3 expression, thereby limiting the infiltration of Th subsets to the site of inflammation.

We have observed differential inflammatory cytokine IFN-γ and IL-4 production by various Th subsets upon TCR stimulation. Although AZM inhibited both IFN-γ and IL-4 cytokine production, the magnitude of IL-4 inhibition in Th0 and Th2-like subset was higher than IFN-γ, suggesting that AZM greatly influences Th2 cytokine IL-4 production, despite its global suppressive effect on Th cytokines. Moreover, significant production of IFN-γ by Th2-like subsets in our experiments perhaps could be due to an incomplete polarization to Th2 effectors as cells were stimulated only for short duration, and in absence of polarizing cytokines. This observation can also be explained by previous studies where TCR-stimulated Th2-like subset shown to acquire higher CCR4 receptor without polarization (18, 25, 32). The cytokine inhibition capacity of AZM was not limited to bulk CD4+ T cell only as FACS-sorted Th0, Th1-like, and Th2-like cells show massive inhibition.

One of the potential mechanisms of AZM mediated CD4+ T cell suppression is through apoptotic induction (10, 16, 17). We also observed similar effects when we tested AZM against TCR stimulated bulk CD4+ T cells. In the same vein, Th subsets also exhibited AZM mediated apoptosis of Th0, Th1-like, and DN subsets. While Th2-like subset showed apoptotic resistance, a feature described by others (33–35). In addition, we also found AZM to significantly reduce the viability of sorted Th1-like, Th2-like, and Th0 subset. Ratzinger et al. has shown a direct effect of AZM on inhibition of mTOR activity as one of the mechanism of CD4+ T cell death (16). Therefore, further investigations are required to understand the involvement of AZM induced mTOR inhibition on Th subsets and other pathways controlling the cell survival.

In this study, our aim was to use bulk CD4+ T cells in order to retain physiologically relevant heterogeneous population and identify Th subsets based on chemokine receptors CCR4 and CXCR3. Although these receptors are well characterized to define Th1 and Th2 subsets (18, 19, 24), their regulation following TCR stimulation, as observed by others (19, 23) as well as our results, may affect the observed phenotype and data interpretation, in particular, the Th expansion results. Perhaps, cytokine driven *in vitro* polarized Th subsets may provide a better model to understand the cell intrinsic AZM effects on these subsets.

Given the contribution of IL-4 in Th2 cell polarization and IgE production, it is possible that beside AZM inhibition of IL-4 production of Th2, Th0 suppression in early stage may limit the polarization capacity of these cells into Th2 effectors. This could be one of the beneficial anti-inflammatory effects attributed to AZM in Th2 dominated airway diseases such as allergy and asthma. The other possibility could be inhibition of inflammatory cytokine producing Th0 subsets infiltration to the site of inflammation as AZM found to reduce the surface CCR4 and CXCR3 expression on these cells. In summary, our findings suggest that AZM exerts differential immunomodulatory effects on Th subsets. Further studies warrant understanding the precise mechanisms of AZM influence on Th subset differentiation into effectors.

## Data Availability Statement

The raw data supporting the conclusions of this article will be made available by the authors, without undue reservation.

## Ethics Statement

The studies involving human participants were reviewed and approved by the Ethics Committee of Dubai Health Authority (DHA) and Dubai Scientific Research Ethics Committee (DSREC-03/2019_10) United Arab Emirates. The participants provided their written informed consent to participate in this study.

## Author Contributions

AA, FS-A, RAH, and QH conceived and designed the experiments. AA, MJ, and AM carried out the experiments. AA, NS-A, and RH performed the data analysis. AA, FS-A, RH, and RAH wrote the manuscript. AM and TV contributed reagents and analysis tools. QH, BM, and RS provided critical inputs to the manuscript. All authors contributed to manuscript revision, read, and approved the submitted version.

## Conflict of Interest

The authors declare that the research was conducted in the absence of any commercial or financial relationships that could be construed as a potential conflict of interest.

## Supplementary Material

FIGURE S1 CD4+ T cell purity. **(A)** FACS plot showing CD4+ T cell purity before (PBMC, left panel) and after (purified CD4+ T cell, right panel) sorting using EasySep separation kit. Cells were stained with anti-CD3 and anti-CD4 antibodies and purity was estimated by flow cytometer. The numbers in quadrant CD3+CD4+ population represent the percent CD4+ T cell purity. **(B)** Gating strategy to characterize helper T cell subsets. Day-3 anti-CD3/CD28 stimulated or unstimulated purified CD4+ T cells were gated on live cells based on FSC and SSC followed by exclusion of doublets. Singlets were gated to characterize CCR4 and CXCR3 based Th subsets. Contour FACS plot shows four distinct sub-population assigned as CCR4+CXCR3- (Th2-like), CCR4+CXCR3+ (Th0), CCR4-CXCR3+ (Th1-like), and CCR4-CXCR3- (double negative, DN) cells. Th subsets were further gated to determine cell proliferation, viability, and cytokine production in respective experiments.

FIGURE S2 AZM suppresses chemotaxis of CD4+ T cells. Anti-CD3 and CD28 stimulated CD4+ T cells were treated with indicated concentration of AZM. On day 3 chemotaxis assay was performed using Transwell system. **(A)** Aligned dot plot show mean ± SEM of the number of cells migrated in response to increasing concentration of CCR4 ligand CCL2 and **(B)** CXCR3 ligand CXCL11. Data presented are from three independent experiments performed on healthy individuals (*n* = 3). ^∗^*P* < 0.05, ns stands for non-significant.

FIGURE S3 Gating strategy for FACS-sorting of helper T cell subsets. Freshly isolated PBMCs were stained with respective monoclonal antibodies and cells were sorted by flow cytometry. PBMCs were gated on live cells based on FSC and SSC followed by exclusion of doublets. Singlets gated cells were subsequently gated for CD3+CD4+ T cells. Sorting was performed by gating on CCR4+CXCR3- (Th2-like), CCR4+CXCR3+ (Th0), CCR4-CXCR3+ (Th1-like) cells.

FIGURE S4 AZM inhibits cytokine production of FACS sorted Th subsets. Freshly isolated around 3–5 × 10^4^ FACS sorted Th subsets were stimulated as described previously with plate-bound anti-CD3 and soluble anti-CD28 in presence or absence of indicated concentration of AZM. Culture supernatants were harvested on day 3 and ELISA was performed. Bar graph show the concentration of IFN-γ **(A)** and IL-4 **(B)**. Data presented are from two independent experiments from two donors. ND denotes non-detectable.

FIGURE S5 PMA/Iono induced cytokine expression. CD4+ T cells were stimulated overnight with PMA (10 ng/ml) and Ionomycin (1 μM). Cells without PMA/iono stimulation were taken as control (US). Cells were labeled with CCR4 and CXCR3 followed by intracellular cytokine staining with anti-IFN-γ and anti-IL-4 mAbs. CCR4+CXCR3- (Th2-like), CCR4+CXCR3+ (Th0), CCR4-CXCR3+ (Th1-like) gated cells were looked at for IFN-γ and IL-4 expression.
