# Supplementary figures and images for: Azithromycin Differentially Alters TCR-Activated Helper T Cell Subset Phenotype and Effector Function
Source: Front Immunol. 2020 Sep 30;11:556579. doi: 10.3389/fimmu.2020.556579 (PMC7575909; doi:10.3389/fimmu.2020.556579)

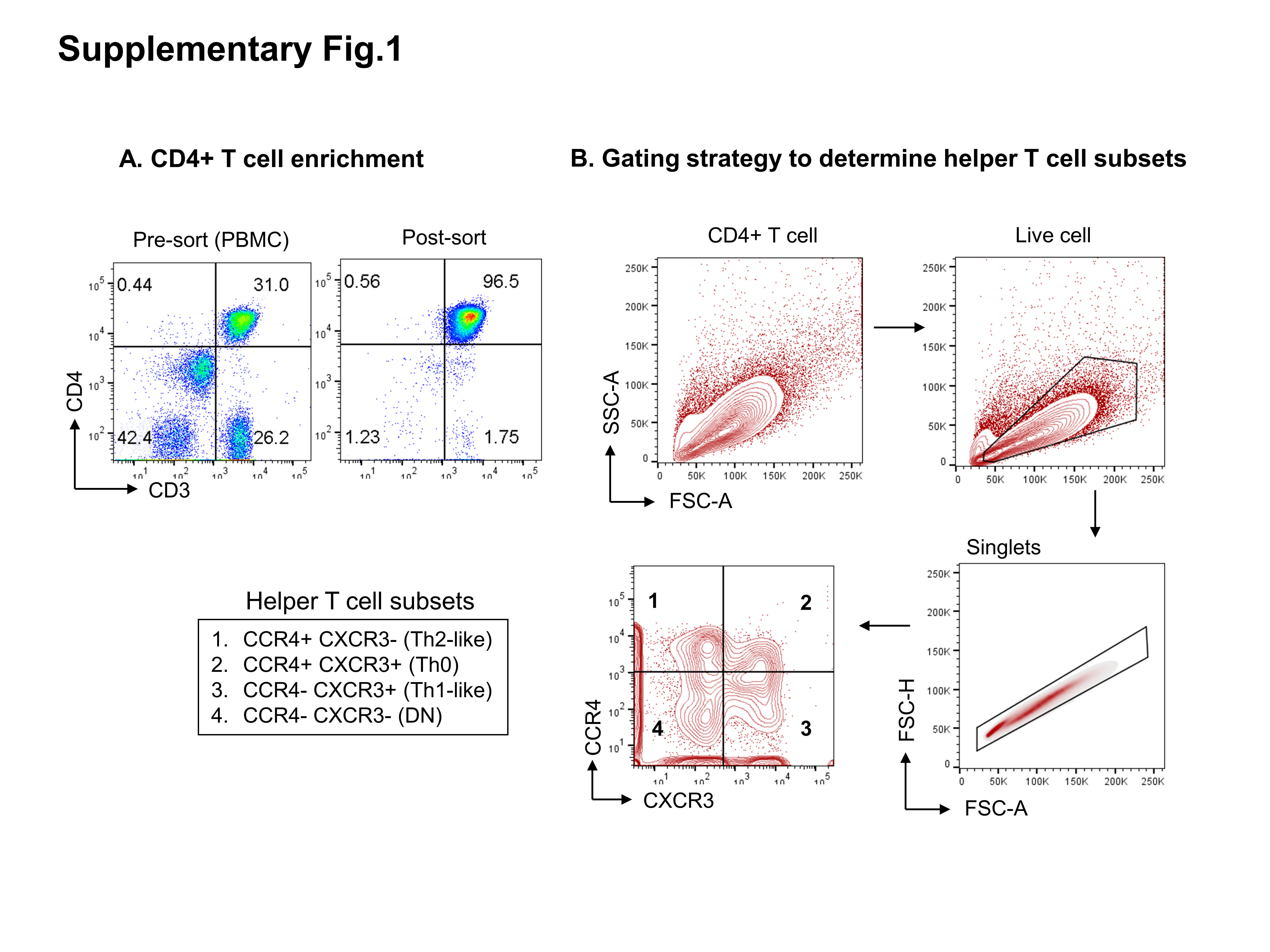

Supplement: FIGURE S1 — CD4+ T cell purity. (A) FACS plot showing CD4+ T cell purity before (PBMC, left panel) and after (purified CD4+ T cell, right panel) sorting using EasySep separation kit. Cells were stained with anti-CD3 and anti-CD4 antibodies and purity was estimated by flow cytometer. The numbers in quadrant CD3+CD4+ population represent the percent CD4+ T cell purity. (B) Gating strategy to characterize helper T cell subsets. Day-3 anti-CD3/CD28 stimulated or unstimulated purified CD4+ T cells were gated on live cells based on FSC and SSC followed by exclusion of doublets. Singlets were gated to characterize CCR4 and CXCR3 based Th subsets. Contour FACS plot shows four distinct sub-population assigned as CCR4+CXCR3- (Th2-like), CCR4+CXCR3+ (Th0), CCR4-CXCR3+ (Th1-like), and CCR4-CXCR3- (double negative, DN) cells. Th subsets were further gated to determine cell proliferation, viability, and cytokine production in respective experiments. [file Image_1.TIF]

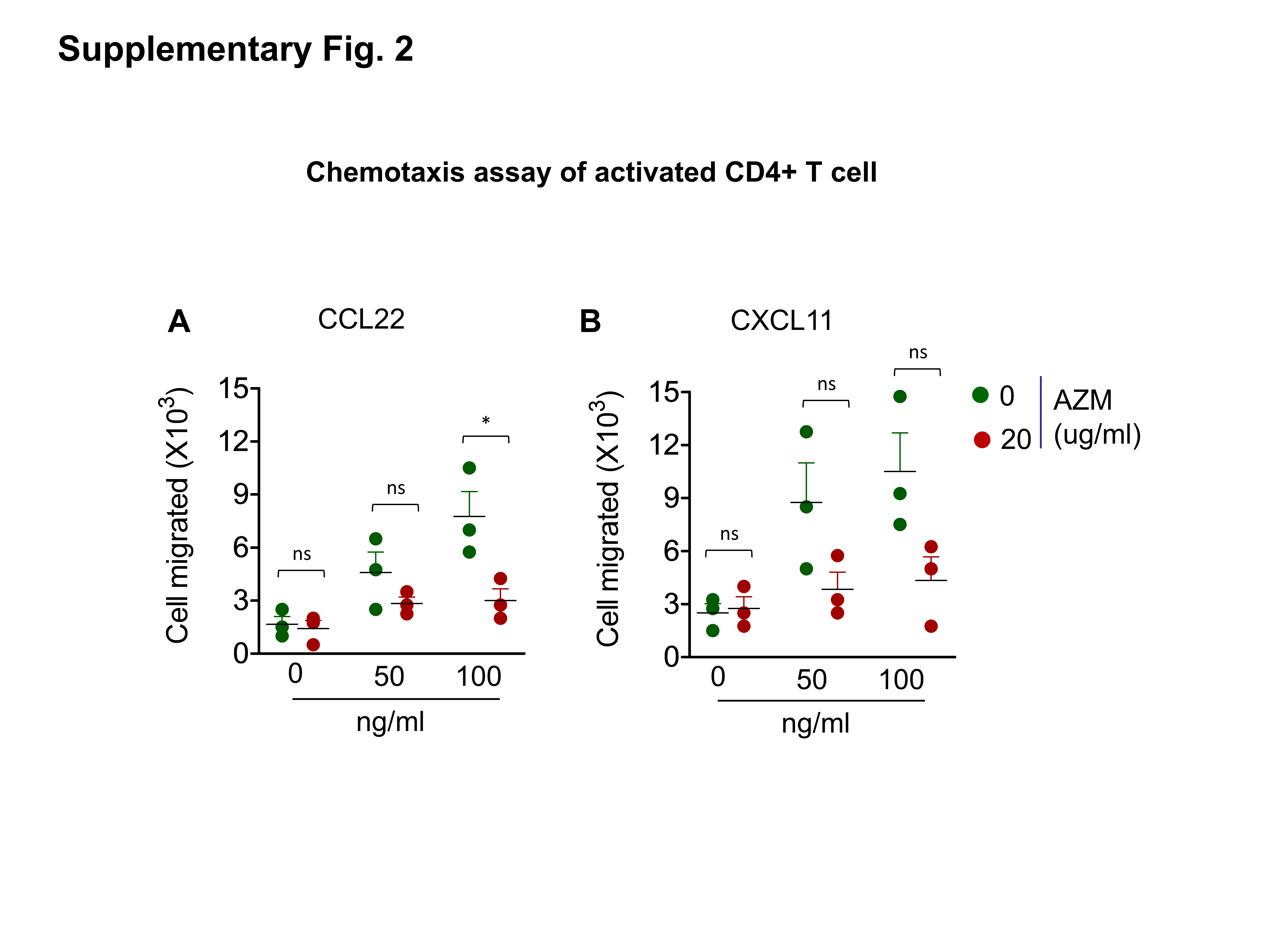

Supplement: FIGURE S2 — AZM suppresses chemotaxis of CD4+ T cells. Anti-CD3 and CD28 stimulated CD4+ T cells were treated with indicated concentration of AZM. On day 3 chemotaxis assay was performed using Transwell system. (A) Aligned dot plot show mean ± SEM of the number of cells migrated in response to increasing concentration of CCR4 ligand CCL2 and (B) CXCR3 ligand CXCL11. Data presented are from three independent experiments performed on healthy individuals (n = 3). ∗P < 0.05, ns stands for non-significant. [file Image_2.TIF]

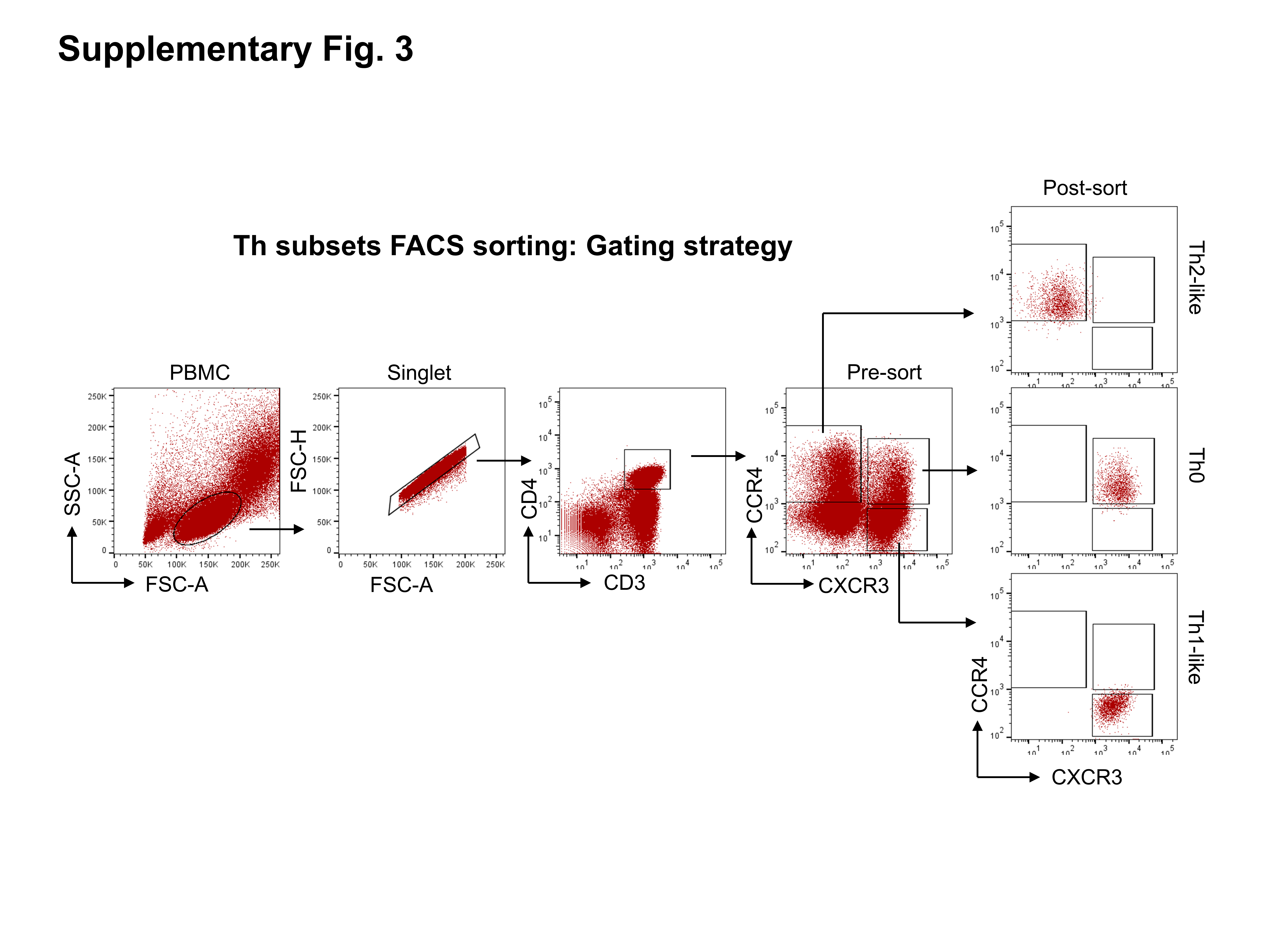

Supplement: FIGURE S3 — Gating strategy for FACS-sorting of helper T cell subsets. Freshly isolated PBMCs were stained with respective monoclonal antibodies and cells were sorted by flow cytometry. PBMCs were gated on live cells based on FSC and SSC followed by exclusion of doublets. Singlets gated cells were subsequently gated for CD3+CD4+ T cells. Sorting was performed by gating on CCR4+CXCR3- (Th2-like), CCR4+CXCR3+ (Th0), CCR4-CXCR3+ (Th1-like) cells. [file Image_3.TIF]

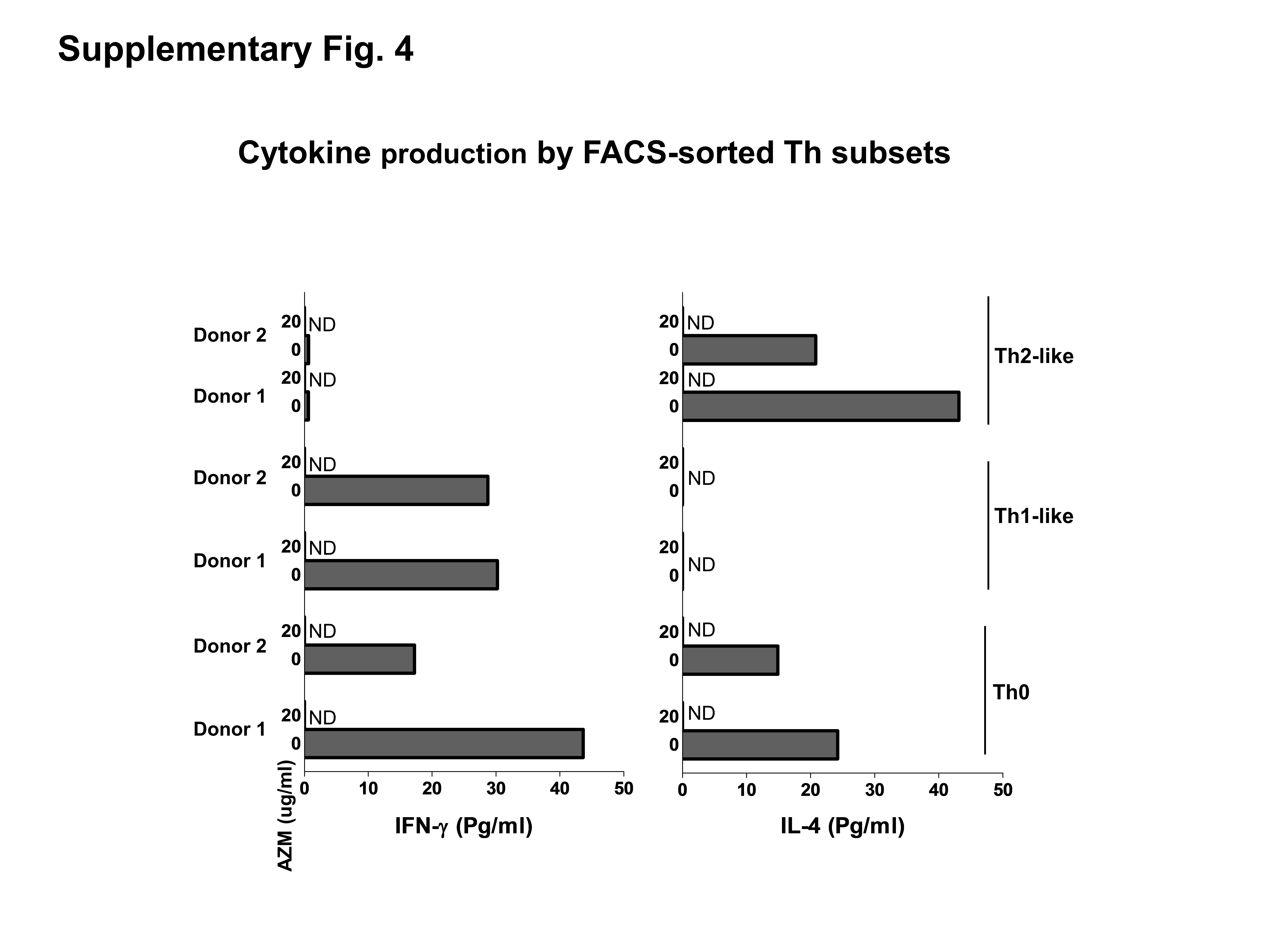

Supplement: FIGURE S4 — AZM inhibits cytokine production of FACS sorted Th subsets. Freshly isolated around 3–5 × 10^4^ FACS sorted Th subsets were stimulated as described previously with plate-bound anti-CD3 and soluble anti-CD28 in presence or absence of indicated concentration of AZM. Culture supernatants were harvested on day 3 and ELISA was performed. Bar graph show the concentration of IFN-γ (A) and IL-4 (B). Data presented are from two independent experiments from two donors. ND denotes non-detectable. [file Image_4.TIF]

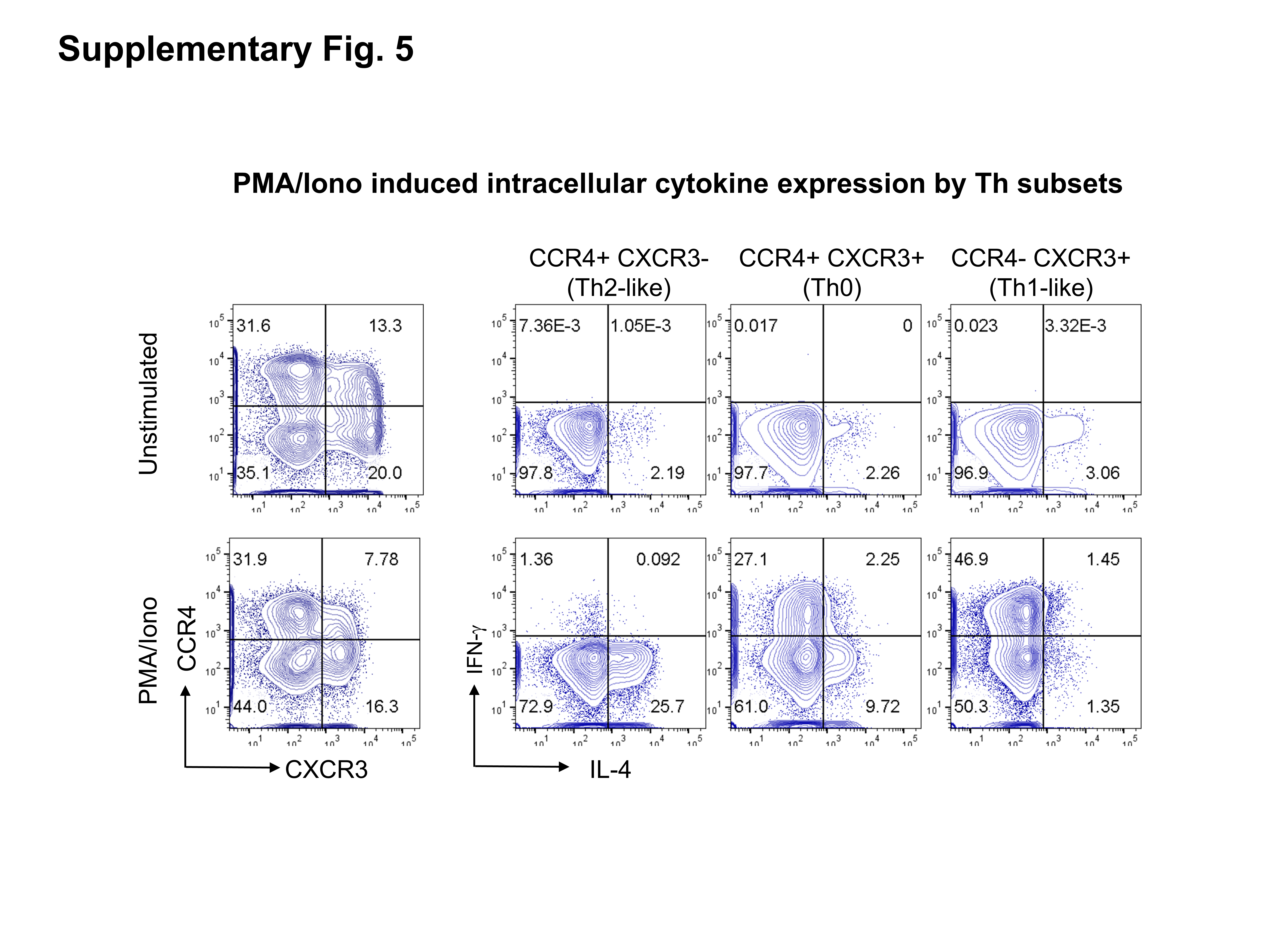

Supplement: FIGURE S5 — PMA/Iono induced cytokine expression. CD4+ T cells were stimulated overnight with PMA (10 ng/ml) and Ionomycin (1 μM). Cells without PMA/iono stimulation were taken as control (US). Cells were labeled with CCR4 and CXCR3 followed by intracellular cytokine staining with anti-IFN-γ and anti-IL-4 mAbs. CCR4+CXCR3- (Th2-like), CCR4+CXCR3+ (Th0), CCR4-CXCR3+ (Th1-like) gated cells were looked at for IFN-γ and IL-4 expression. [file Image_5.TIF]
